# 1391. Association between Substance Abuse and Mental Illness Symptoms Screener (SAMISS) scores and HIV care cascade outcomes in newly diagnosed people with HIV (PWH) in the US South

**DOI:** 10.1093/ofid/ofac492.1220

**Published:** 2022-12-15

**Authors:** Manal Ahmed, Ank E Nijhawan, Jeremy Y Chow, Ang Gao, Chul Ahn

**Affiliations:** UT Southwestern, Irving, Texas; UT Southwestern, Irving, Texas; UT Southwestern, Irving, Texas; UT Southwestern, Irving, Texas; UT Southwestern, Irving, Texas

## Abstract

**Background:**

Mental illness (MI) and substance use (SU) among people with HIV (PWH) impact multiple stages of the care cascade. Little is known about baseline mental illness and substance use as defined by the Substance Abuse and Mental Illness Symptoms Screener (SAMISS), a common tool used at entry into care, and subsequent care cascade outcomes.

**Methods:**

A retrospective chart review was conducted of individuals newly diagnosed with HIV between 2016-2019 in an urban HIV clinic in Dallas, Texas. Baseline demographic, HIV labs, socio-economic variables and availability of rapid ART (implemented 10/2018) were collected. Patients were screened for MI and SU using the SAMISS. Outcomes included retention in care (2 encounters within 12 months, 90 days apart), probability of achieving virologic suppression (VS = viral load (VL) ≤200 copies/mL), and sustained VS at 12 months. Data analyses included stepwise Cox and logistic multivariate regression modeling conducted in SAS, version 9.4. Covariates with p< 0.2 in univariate analyses were entered as candidate variables for stepwise selections.

**Results:**

Among the 530 newly diagnosed PWH, mean age was 35, 79% were male, 46% non-Hispanic Black, 37% Hispanic. Overall, 34% screened positive for SU and 41% for MI; 46% received rapid ART. In univariate analyses, those screening MI-positive were less likely to be retained in care (OR 0.68, p=0.035), or to have continuous VS (OR 0.61, p=0.005), though neither remained significant once controlling for rapid ART initiation and other covariates. In univariate analysis, those screening SU-positive were less likely to achieve VS (HR 0.80, p=0.030), and in multivariate analyses, they were less likely to be retained in care (OR 0.60, p=0.015) or to have continuous VS (OR 0.61, p=0.015) after adjusting for rapid ART initiation and covariates.

**Conclusion:**

In a safety-net southern US clinic, baseline SAMISS MI and SU scores were associated with worse 12-month retention in care and virologic suppression. Given widespread adoption of rapid ART initiation soon after HIV diagnosis, further studies should explore how initial assessment scores like SAMISS could be used to both identify and direct interventions to at-risk, newly diagnosed PWH to impact long-term retention and VS.

**Disclosures:**

**Ank E. Nijhawan, MD, MPH, MSCS**, Gilead Sciences: Grant/Research Support **Jeremy Y. Chow, M.D., M.S.**, Gilead Sciences: Grant/Research Support.

